# “I just held it to myself”: screening and treatment experiences of individuals with perinatal suicidal thoughts and behaviors

**DOI:** 10.1186/s12888-026-08129-3

**Published:** 2026-05-02

**Authors:** Lauren A. Kobylski, Alex A. Schroeder, Catherine K. Thong, Jennifer M. Keller, Huynh-Nhu Le

**Affiliations:** 1 Department of Psychological & Brain Sciences, George Washington University, 2013 H St NW, Washington, DC 20006 USA; 2 Department of Obstetrics & Gynecology, The George Washington University School of Medicine and Health Sciences, 2300 I St NW, Washington, DC 20037 USA

**Keywords:** Perinatal, Maternal, Suicide, Suicidal thoughts and behaviors, Screening, Treatment

## Abstract

**Background:**

Suicide is a leading cause of maternal mortality, yet there are currently no evidence-based perinatal suicide prevention programs. Given the risk of serious outcomes if undetected or inadequately treated, the goal of this study was to further understand the screening and treatment experiences of individuals with perinatal suicidal thoughts and behaviors (STBs).

**Methods:**

Qualitative data were generated from in-depth interviews with 13 individuals primarily from the United States who experienced perinatal suicidality at least 6 months prior to participation. Thematic analysis was used to examine the experiences of participants with respect to screening and treatment of perinatal STBs.

**Results:**

Regarding screening, three major themes were identified: (1) *gaps in comprehensive/routine screening for STBs* (e.g., infrequent screenings or non-specific to suicide), (2) *attitudes toward disclosure of STBs* (resulting in omission of symptoms or downplaying of severity), and (3) *importance of follow-up after screening*. Three themes influenced participants’ treatment experiences: (1) *providers’ engagement in care*, (2) *shared decision-making between provider and patient*, and (3) *impact of perinatal-specific treatment programs*.

**Conclusion:**

Findings from this study highlight critical gaps in screening for and treatment of perinatal STBs. Implementing routine screening and comprehensive follow-up and improving treatment experiences are essential for improving the care of individuals with perinatal STBs and reducing maternal mortality.

**Supplementary Information:**

The online version contains supplementary material available at 10.1186/s12888-026-08129-3.

## Background

Suicide is a leading cause of maternal mortality in the United States (U.S.) [1–3]. Globally, up to 20% of postpartum deaths are due to suicide [4]. Rates of perinatal suicidal ideation (SI) are also concerning, estimated at 8% globally [5]. In the U.S., rates of SI during pregnancy and postpartum have risen over time, and with certain populations experiencing the sharpest proportional increases (e.g., Black birthing individuals, individuals with lower incomes, younger individuals) [6, 7]. Perinatal suicidal thoughts and behaviors (STBs) are also associated with multiple adverse consequences for mothers, their children, and mother-infant relationships [8–12]. If reduction of maternal mortality is to be achieved, a focus on suicide in the perinatal period (i.e., pregnancy and the first year postpartum) is of critical importance. However, there are currently no evidence-based psychological interventions to prevent perinatal suicide [13, 14]. There is a pressing need for research examining how individuals are currently being screened and treated for perinatal STBs in order to develop effective interventions.

Screening practices for perinatal mental health concerns and STBs vary considerably across countries. Within the U.S., the landscape is similarly heterogeneous. Multiple professional organizations, including the American College of Obstetricians and Gynecologists and U.S. Preventive Services Task Force, recommend screening for depression and anxiety symptoms at least once during the perinatal period using a validated tool [15–17]. However, no federal mandate for universal perinatal mental health screening exists in the U.S., nor are there currently any professional guidelines recommending standardized screening for perinatal STBs, leaving implementation variable based on the state, healthcare system, and provider. Crucially, existing screening tools vary in the degree to which they assess STBs, with many focusing on depressive symptoms with only a singular item assessing STBs [18]. Following a positive suicidality screening or disclosure of STBs, referral and treatment pathways are similarly diverse. U.S.-based perinatal individuals may be provided with a referral to outpatient therapy, a prescription for psychiatric medication, or in more acute cases, a referral to higher levels of care. Like screening, these pathways are largely dependent on the health system and the provider’s training and experience, and access to perinatal-specific mental healthcare remains limited across the country. Additional structural features of the U.S. healthcare system (e.g., insurance-based healthcare access, fragmentation between obstetric and mental healthcare, shortages of providers with expertise in perinatal mental health) likely compound these issues. Qualitative research is a valuable methodology for understanding lived experiences and informing patient-centered health research. A small but growing body of qualitative literature has illuminated the complex phenomena of perinatal STBs, clarifying numerous psychological, social, and systemic contributors. Two studies, respectively originating from the United Kingdom (U.K.) and Australia, utilized a grounded theory approach to understand the origins of perinatal suicidality [19, 20]. Reid et al. (2022) identified the concept of being “attacked” by motherhood (e.g., loss of control, isolation, unmet expectations of motherhood, uncomfortable feelings toward the baby) as being central to eventual negative self-evaluation as a mother, which leads to difficulty coping, perceptions of failure, entrapment, and ultimately, suicidal thoughts. Similarly, Biggs et al. (2023) explicated a psychosocial trajectory tracing the impact of adverse childhood experiences, violated expectations of motherhood, and loss of identity, culminating in loss of control, isolation, and eventually, suicidality. Other work from the U.K. has similarly emphasized the cumulative weight of trauma, life stressors, and disillusionment with motherhood in leading to despair and eventually attempted perinatal suicide [21]. Other qualitative work on perinatal STBs has highlighted the influence of interpersonal and structural factors. In Kenya and South Africa, interpersonal violence, family rejection, community stigma, and poverty and income inequality were identified as contributors to suicidal behaviors [22, 23].

Despite this growing understanding of contributors to perinatal STBs, less is known about perinatal individuals’ experiences with screening of and treatment for STBs. Recently, a team in the U.K. queried 21 perinatal women (with and without experiences of perinatal mental health problems) regarding their perceptions of suicide screening items and attitudes toward disclosure of suicidality in healthcare settings [24, 25]. Qualitative analysis revealed that participants found standard screening language for perinatal STBs to be alienating, stigmatizing, or confronting [24]. Multiple salient barriers to disclosure of suicidality were identified, including stigma, shame, societal expectations of motherhood, and fearing the consequences of disclosure; these barriers were especially prominent when coupled with rushed screenings, minimization of symptoms by healthcare providers (HCPs), and a perceived emphasis on the baby’s well-being over that of the mother [25]. To our knowledge, no studies to date have examined qualitative experiences of treatment for perinatal STBs.

Even when perinatal STBs are disclosed, the process of managing emotional distress can be challenging. To the best of our knowledge, only one qualitative study of perinatal STBs has been conducted in the U.S. In this work, screening was sometimes seen as an opportunity to signal distress to HCPs, and other times, relational and contextual factors shaped whether women felt they could speak openly about SI. While some participants described positive coping strategies (e.g., seeking support, relying on religion/spirituality), others turned to maladaptive strategies, including self-harm, substance use, or social withdrawal [26].

These studies, taken together, have provided rich insights into the psychological, social, and structural roots of perinatal STBs, and have revealed a preliminary understanding of perinatal individuals’ perceptions of screening for suicide risk. However, critical gaps remain, particularly with respect to lived experiences of treatment for perinatal STBs. Further, the vast majority of available studies are not based in the U.S., where access to and types of healthcare are markedly different than in other parts of the world. The present study seeks to address this gap by qualitatively exploring the screening and treatment experiences of individuals with experiences of perinatal STBs in a primarily U.S.-based sample. A greater understanding of these topics has the potential to inform future prevention and intervention efforts, facilitate recovery, and improve outcomes for perinatal individuals and their families.

## Methods

### Study design

The Perinatal Experiences and Assessment of Recovery from Suicidality (PEARS) study utilized an exploratory qualitative design to examine various aspects of the lived experiences of perinatal suicidality, including onset and symptoms, coping strategies and support, screening and treatment experiences, and intervention preferences. The overarching aim of the PEARS study is to generate knowledge directly from the voices of those affected by perinatal STBs to improve perinatal suicide prevention efforts. Qualitative methods are especially useful in examining sensitive topics, like STBs, and can facilitate greater insight into the topic of inquiry than quantitative methods alone [27].

**Fig. 1:**
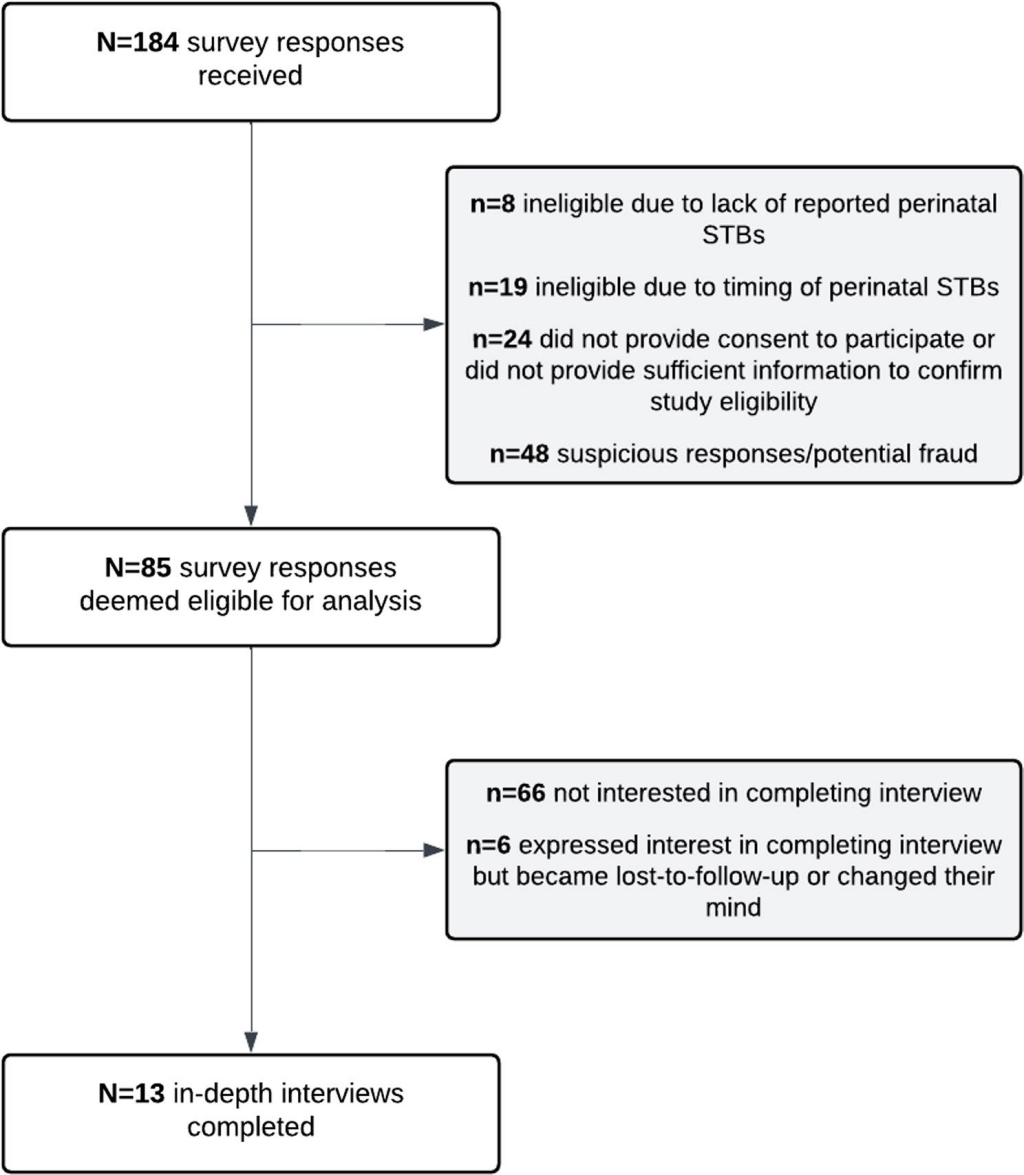
Study enrollment. Note. STBs = suicidal thoughts and behaviors

Individuals who were eligible to participate in this study first completed an online survey including written informed consent, basic demographic information, and a few open-ended questions regarding their experiences. If interested, participants who completed the survey were entered into a raffle for $25 gift cards. Those who were interested in completing a follow-up in-depth interview indicated their willingness to do so in this survey. Interview completers were compensated with a $40 gift card for their time. Study enrollment is depicted in Fig. 1. 85 analyzable survey responses were received, and 13 interviews were completed during the study timeframe. The present analysis draws upon data generated from the in-depth interviews.

### Participants and recruitment

In order to ensure a diversity of experiences was included, eligibility criteria for the PEARS study were minimal. Eligible participants were 18 years or older, could speak and write English, and self-reported an experience of perinatal suicidality at least six months prior to participation. This timeframe was selected to increase the likelihood that participants were psychologically stable at the time of participation and that their involvement would not pose threats to their emotional safety.

Recruitment occurred through multiple avenues. The PEARS study was advertised through social media and through the websites, newsletters, and social media accounts of various nonprofit organizations (e.g., Postpartum Support International, Suicide Awareness Voices of Education). Flyers were also disseminated in local obstetric and primary care clinics. Recruitment occurred between April 2024 and May 2025, concluding when thematic saturation was achieved (i.e., when no additional insights were identified during interviews, and when interview content began to repeat itself).

### Data collection and analysis

Thirteen interviews were conducted virtually by the first author (LAK), lasting between 41 and 108 min (*M* = 83). The interview guide explored experiences of perinatal STBs across multiple domains, including emotional experiences, mental health symptoms, coping resources and support, screening and treatment, and preferences for future interventions. The semi-structured format allowed participants to flexibly share their story with prompting and guiding by the interviewer. Interviews were audio-recorded and transcribed by two members of the study team (AAS, CKT). All transcripts were double-checked for accuracy and to ensure complete de-identification.

Reflexive thematic analysis of the interview data focusing on the screening and treatment experiences of participants was undertaken using ATLAS.ti Web (version 9.18.0) [28]. Specifically, Braun and Clarke’s six-phase process was used as a guideline to identify key themes regarding individuals’ lived experiences of screening and treatment of perinatal STBs [29, 30]. The study team began by familiarizing themselves with the transcripts and making notes about their observations of participant narratives. A codebook was generated both inductively from the data and deductively from interview topic areas. Two coders (AAS, CKT) applied codes to the transcripts with guidance from the first author of this paper (LAK), who has experience and training in qualitative methods. All coded transcripts were reviewed at least twice to ensure comprehensiveness. The codebook was revisited and revised iteratively throughout the coding process. Meetings occurred regularly between members of the coding team to consider code application, resolve discrepancies, and discuss findings and personal reactions. After completion of coding, data displays were constructed to organize and group related codes into broader categories. Themes were revised until agreed upon by all coders and the first author. All participants were invited to review the final themes and manuscript and provide feedback (i.e., member checking).

### Reflexivity

The study team capitalized on reflexivity throughout the research process, and professional and personal experiences were proactively drawn on to work through methodological and contextual issues [31, 32]. Backgrounds of the study team members varied, including those with clinical training in evidence-based psychological interventions (LAK, HL) and obstetrics and gynecology (JK). The team was balanced between those with and without children and personal histories of mental health challenges. Most team members were familiar with the phenomenon of perinatal mood and anxiety disorders and had varying levels of knowledge regarding STBs and suicide prevention. Interviews were conducted by a clinical psychology doctoral candidate with research training in qualitative methods and clinical training in crisis intervention and perinatal mental health (LAK). Analysis was led by this same researcher alongside two other study team members who had undergraduate backgrounds in psychology (AAS, CKT). Thus, the team was well-positioned to engage critically with participant narratives, bringing clinical insights and personal experiences to the analysis, while remaining attentive to the impacts of positionality on interpretations.

### Ethical considerations

All study procedures were approved by the George Washington University Institutional Review Board. Verbal informed consent was obtained from all participants by the first author (LAK) prior to initiating the interviews. Given the sensitive nature of the topics discussed in the PEARS interview, a robust protocol including risk assessment, safety planning, and referral to emergency resources was developed to ensure participant safety. It is important to note that while some participants expressed increased emotionality while sharing their story (e.g., grief, frustration), there were no incidents of active STBs or increased suicide risk such that the safety protocol was deployed.

## Results

**Fig. 2:**
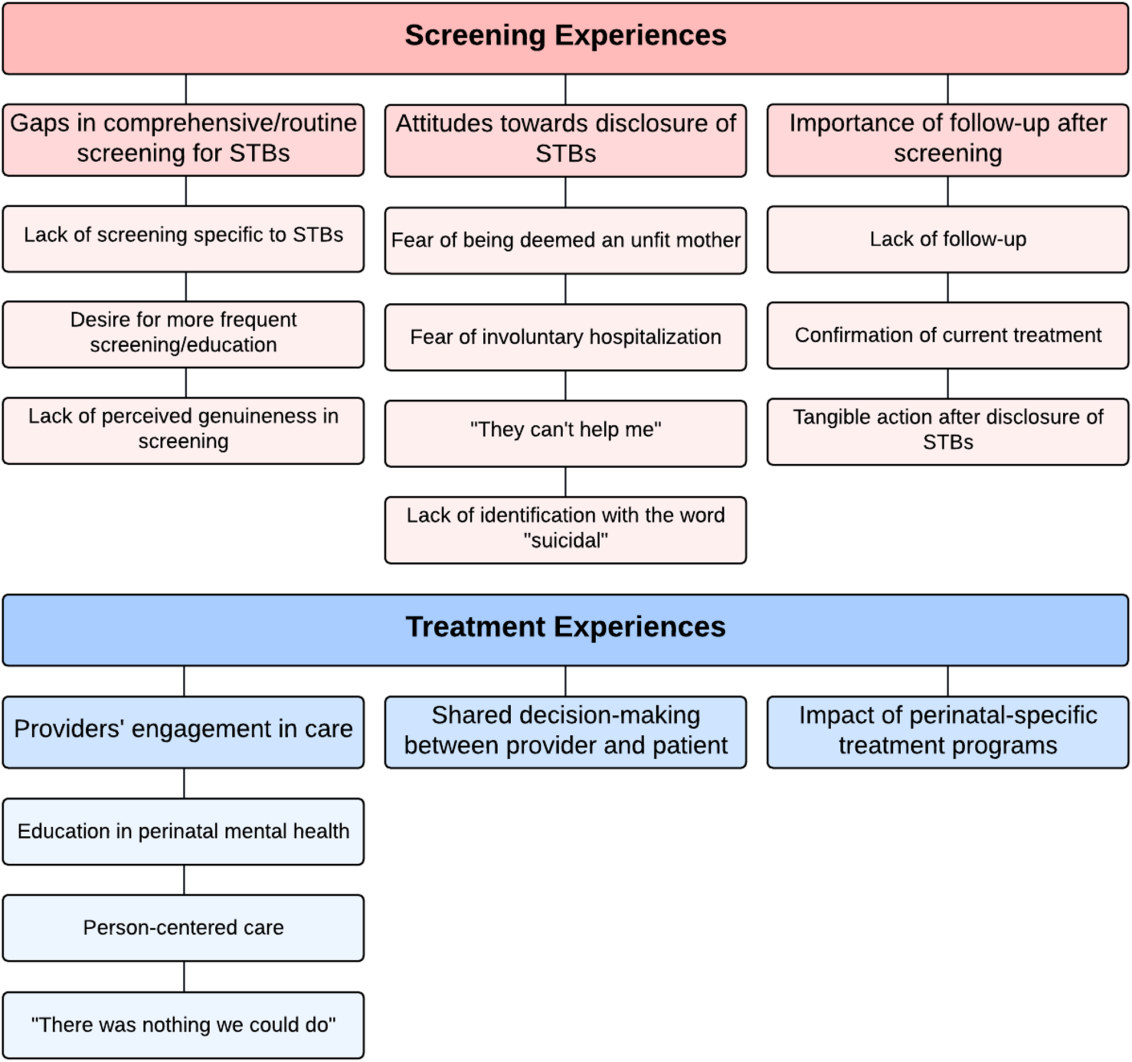
Overview of study themes. Note. STBs = suicidal thoughts and behaviors

Sociodemographic characteristics of the sample are displayed in Table 1. About half of the sample identified as White, and nearly one-third identified as Black; the average age was 29.31 (*SD* = 4.84). The majority of participants were U.S.-based, married, and college-educated at the time of their experience with perinatal STBs. Participants described a range of experiences on the STB spectrum, ranging from a lack of desire to live, active SI with a plan, to aborted suicide attempts. A variety of treatment types were received, including individual and group therapy, medication, and higher levels of care (i.e., intensive outpatient program (IOP), inpatient hospitalization). Key themes related to the screening and treatment experiences are discussed under the corresponding headings and presented in Fig. 2. Illustrative quotes from participants are included throughout.

**Table 1:** Sample characteristics (*N* = 13)

Characteristic		*n* (%)
Race	Asian	1 (7.69)
	Black/African American	4 (30.77)
	White/Caucasian	7 (53.85)
	Prefer not to answer	1 (7.69)
Ethnicity	Non-Hispanic	12 (92.31)
	Hispanic	1 (7.69)
Country of Birth	United States	12 (92.31)
	Canada	1 (7.69)
Lifetime Number of Pregnancies	1	4 (30.77)
	2	7 (53.85)
	5+	2 (15.38)
Relationship Status^a^	Married	11 (84.62)
	Partnered	1 (7.69)
	Divorced/separated	1 (7.69)
Educational Level^a^	High school diploma or GED	1 (7.69)
	4-year college degree (i.e., Bachelor’s)	6 (46.15)
	Graduate degree	6 (46.15)
Neighborhood Type^a^	Suburban	9 (69.23)
	Urban	4 (30.77)
Country of Residence^a^	United States	12 (92.31)
	Canada	1 (7.69)
Timing of Perinatal STBs	Pregnancy only	1 (7.69)
	Postpartum only	8 (61.54)
	Both pregnancy and postpartum	4 (30.77)
		***M*** **(*SD*)**
Age^a^ (years)		29.31 (4.84)
Interview Length (minutes)		83.19 (18.01)

^a^Assessed at time of experience with perinatal STBs

Note. GED = General Educational Development. STBs = suicidal thoughts and behaviors

### Screening experiences

Regarding participants’ experiences of screening for perinatal STBs, three major themes were identified: (1) *gaps in comprehensive/routine screening for STBs*, (2) *attitudes towards disclosure of STBs*, and (3) *importance of follow-up after screening*.

### Gaps in comprehensive/routine screening for STBs

This theme reflects participants’ experiences with screening processes largely perceived as inadequate or insufficient. Sub-themes include: (1) *lack of screening specific to STBs*, (2) *desire for more frequent screening/education*, and (3) *lack of perceived genuineness in screening*.

#### Lack of screening specific to STBs

Not being screened specifically for STBs during the perinatal period was common among study participants. The majority expressed disappointment and/or frustration around this lack of screening, and a desire for specific screening questions regarding STBs and perinatal mental health challenges (rather than broad questions around general well-being). For instance, one participant shared that her mental health challenges would have been identified earlier with specific screening tools, like the Edinburgh Postnatal Depression Scale (EPDS; [33]). Instead, she received a non-specific, general mental health check-in, during which it was easier to lie.

*I didn’t even get the screener. He asked me*,* ‘How are you feeling? Are you feeling okay mentally? Anything that you’re struggling with?’ and I just lied. I said*,* ‘Yeah*,* I’m feeling fine.’ ‘Cause they didn’t make me fill out the Edinburgh or anything like that…I think if they were trained to ask more specific questions rather than a general umbrella of*,* ‘How are you doing*,* are you feeling okay?’ *[1]

Being asked about STBs specifically during mental health screenings was perceived to facilitate disclosure of STBs and decrease feelings of shame surrounding initiating such conversations.

#### Desire for more frequent screening/education

Participants felt that they were not screened frequently enough during the perinatal period, particularly in the vulnerable postpartum period.

*I think better—more thorough—postpartum care for the mom would have prevented me from getting that bad. If I had been meeting with my doctors regularly postpartum*,* if I had been given screeners postpartum*,* if I had been given some education about PMADs* [perinatal mood and anxiety disorders]*…it would have been preventable*,* or at least preventable in the fact of not getting that bad.* [1]

Alongside screening, participants wanted more education on perinatal mental health during appointments. Such proactive education on specific signs and symptoms was identified as an opportunity to facilitate early identification of and treatment for STBs.

*…If there was more of a focus in the prenatal period about mental health in the postpartum*,* I think that too*,* would’ve helped me*,* instead of being like*,* ‘Oh yeah*,* this is a thing that can happen*,* let us know if it does.’ If it was like*,* ‘Hey*,* this is a thing that can happen*,* this is what it looks like*,* this is about when women start to experience it*,* these are the people that you can reach out to”* or *“Let’s get you on their schedule just in case*,* —you can always cancel the appointment if you don’t need it.’ But more education about what it looks like*,* how it can feel generally*,* versus the ‘wait and see’ approach*,* would be so much better.* [12]

#### Lack of perceived genuineness in screening

Many participants felt that their providers were screening for mental health concerns because they were required to, rather than possessing a genuine concern about their well-being. When clinicians were perceived as insincere, participants believed that it would be more tiring, time-consuming, and emotionally draining to disclose and discuss STBs with providers than to cope with them on their own.

*They would ask about it*,* but—I’m like*,* okay*,* do you really care…Like a checked box…The OB that I had immediately postpartum*,* I never met before…and I felt like when she asked*,* I was navigating*,* a toddler*,* a newborn*,* a C-section recovery. And when she asked me*,* I was like*,* alright…you’re asking that ‘cause you have to…I just don’t feel that you’re very genuine*,* and I am not going to waste my time trying to make you feel genuine towards me…I would just be like*,* ‘No*,* I haven’t*,* it’s fine*,* move on.’ It’s just easier for me to not even engage in that conversation with you than it is to just take care of it myself.* [12]

### Attitudes towards disclosure of STBs

This theme reflects the contributors to participants’ decision of whether or not to share STBs with providers. Sub-themes include: (1) *fear of being deemed an unfit mother*, (2) *fear of involuntary hospitalization*, (3) *“they can’t help me”*, and (4) *lack of identification with the word “suicidal.”*

#### Fear of being deemed an unfit mother

Most participants expressed a fear of being deemed an unstable or incompetent mother if they divulged their STBs to an HCP. For instance, one participant decided not to disclose her STBs to prevent a potential separation between her and her child.

*[It] would’ve been more helpful if I were honest about it…At that point*,* I had already kind of spiraled into the “not feeling good” state*,* and I didn’t want her to take away [my child] and have us separated…So*,* it was very easy for me to just be like*,* ‘Nope*,* I’m doing great*,* actually*,* you know—just kind of trucking along.’ And once I had said that*,* the conversation just kind of ended at that point. *[4]

Further, some participants perceived questions about STBs to be asked solely for the purpose of identifying unfit mothers, as opposed to an opportunity to provide support.

*I put all the depression symptoms. When it said*,* ‘Do you think of harming yourself?’ No…If I felt like I would just be given the help that I needed*,* then I would do it. Before that question is even asked on a form*,* if that help was available*,* it would’ve been offered to me. So*,* I’m like*,* okay*,* there is no help. You’re asking me this because you think I’m just going to kill myself and my baby*,* and you want to take them away from me before it’s done. I want the help*,* but I’m afraid you’re not going to give it to me…Once people feel like they have to identify themselves*,* and they’re growing a human inside that they’re afraid they’ll lose—I think that causes a lot of people just not to say anything…*[7]

#### Fear of involuntary hospitalization

Participants feared that disclosing the presence or severity of their STBs would lead to involuntary hospitalization. One participant disclosed their SI to their therapist, but not their level of intent and plan.

*You want to talk to your therapist*,* but you don’t want to tell them so much that they hospitalize you. So*,* I did tell her I’d had ideation*,* but I don’t think I ever told her*,* you know*,* ‘I really*,* I’m about to do this.’* [7]

Some participants worried that providers would “panic” following a disclosure of STBs and immediately direct them to an inpatient hospitalization, rather than first trying lower levels of treatment (e.g., outpatient therapy, medication). Further, some felt that hospitalization would not have been the most effective treatment for them at that time. As a result of these fears, one participant downplayed her self-harm ideation to her providers.

*My fear was that she was going to see those [screening] results and…someone was gonna fucking commit me*,* because they were bad… I felt that going inpatient at that exact moment was going to be worse*,* because I was not a danger to myself. I did not have a plan. I did not have anything that was going to end my life*,* and I was worried that they would jump straight to the end of the line before trying therapy*,* before trying meds… I downplayed the thoughts of harming myself*,* mostly because I knew that I wasn’t going to kill myself*,* but people get very panicky when you say like*,* ‘I cut myself.’ And they’re like*,* ‘Oh my God! We gotta commit you!’* [13]

Participants appreciated when their HCPs took the time to understand and explore their concerns, rather than immediately recommending hospitalization; for instance, participant 9 shared, “…*my therapist has been really supportive…she didn’t just jump to*,* ‘Okay*,* you need to go to the hospital*’*—just kind of exploring and asking questions.*”

#### “They can’t help me”

Some participants chose not to disclose STBs during screening because they doubted their providers’ ability to support them meaningfully. When participants perceived their mental health as too severe for non-specialists to address, disclosing STBs to someone without the proper expertise felt pointless.

*Yeah*,* they did [ask about STBs]*,* but I didn’t just want to tell them what I was going through. I just held it to myself…I just felt like*,* they can’t help me*,* or they were busy*,* or they would even worsen my case.* [1]

Several participants experienced negative or harmful experiences with providers and broader systems of care (explored further in the domain of treatment experiences), contributing to a sense of hopelessness regarding providers’ ability to support them or connect them to appropriate resources. As a result, being honest during screenings regarding STBs was viewed as pointless and even potentially harmful. This is reflected in the narrative of one participant, whose treatment team’s lack of support in navigating active domestic violence and associated legal challenges influenced her attitude toward STB disclosure.

*I really felt so unsupported through the delivery process and…the hospital not having notes*,* and that really impacting the [temporary restraining order]—it was like*,* ‘You guys haven’t helped in a year*,* I don’t think you’re going to be helpful now.’* [5]

#### Lack of identification with the word “suicidal”

While a less frequent phenomenon in this sample, lack of identification with the word “suicidal” was a salient reason for denying the presence of STBs during screening. Some perceived the term as too intense or stigmatizing, and therefore not directly applicable to their experiences.

*I think that if they had asked me*,* ‘Are you experiencing suicidal thoughts?’*,* I would’ve said no*,* anyways. I think the wording of that is not where my brain was. But if they had asked me*,* ‘Have you had thoughts of ending things*,* or escape?’*,* that would’ve clued me in…But like suicidality*,* suicide—I mean*,* that’s such a buzzword…Especially growing up in the early 2000s when…there was such a culture about suicide being so bad and evil…So*,* I don’t think I would’ve clued in that*,* yes*,* that’s what I’m feeling and that’s what I am thinking…because of that internalized shame around it.* [8]

Thus, some participants did not identify themselves as being suicidal due to a disconnect between their own perceptions of their STBs and the direct language used in screening as well as cultural norms and stigma surrounding suicide.

### Importance of follow-up after screening

This theme reflects the wide variation in participants’ experience of follow-up after STB screening. Experiences were organized into the following sub-themes: (1) *lack of follow-up*, (2) *confirmation of existing treatment*, and (3) *tangible action after screening*.

#### Lack of follow-up

Following a positive STB screening or endorsement of STBs, many participants felt that their providers failed to provide clear direction to appropriate resources or treatment options, leading to frustration and a sense of being alone in navigating their care.

*I did see in the bathroom they had a thing hanging up. I think it said*,* ‘A percentage of women experienced depression during pregnancy.’ And I was like*,* ‘What? Oh!’ Nobody actually asked me*,* but I did say it to them. But when I said it to them*,* they were like*,* ‘Oh no…We put the information up*,* and now here’s one. Now what do we do with it? Somebody actually came!’…They didn’t have the knowledge of what to do.* [7]

Reflecting on inadequate follow-up experiences, participants communicated a desire for clear, consistent guidance and proper direction from their providers in navigating treatment.

*…When I look back*,* there was so much time wasted on me beating myself up*,* and digging my own grave in a way*,* that if somebody had just stepped in…if I had been automatically on somebody’s schedule right off the bat*,* even if I felt fine*,* I wish that I was made to see that person on a weekly basis…that early intervention would have made me feel so much more seen*,* and I would’ve had so many more things explained to me—that how I was feeling at that time was normal. Versus like*,* letting it fester and then it became what it was…* [12]

Overall, when follow-up after screening was perceived as minimal and when providers appeared to lack knowledge on available resources or dismissed participants’ concerns, participants described a sense of being unsupported and left alone to find effective treatment options.

#### Confirmation of current treatment

Several participants felt that their endorsement of STBs during screening were dismissed once providers confirmed that they were already receiving some form of mental health support.

*[My doctor] asked if I had a psychiatrist*,* or any mental healthcare*,* counselor*,* anything like that*,* and I told him I did already have that care. He’s like*,* ‘Okay*,* then we’re good here.’ *[11]

Participants’ experiences suggested that, from the perspective of obstetric providers, confirmation of existing mental health treatment was sufficient in addressing STBs.

*It’s possible I did [fill out a screener]…and then they were like*,* ‘Oh yeah*,* we already know you have a therapist*,* we already know you have a psychiatrist—you’ll be fine.”…There wasn’t a lot of checking in about it. Even if they knew*,* maybe they just felt ‘the therapist probably has it under control.’* [3]

Participants reported that these conversations ended abruptly after current treatment was confirmed, hindering an opportunity for obstetric providers to offer additional guidance, resources, or meaningful follow-up.

#### Tangible action after disclosure of STBs

Participants were most satisfied with their experience of STB disclosure when providers responded with tangible action (e.g., prescribing medication, facilitating connections with additional HCPs to coordinate care).

*The first time I went to the hospital was for those*,* like*,* self-harm thoughts…I was able to text my OB and tell him—this is what is happening*,* I’m going to go to the hospital. So*,* he put a call in to let them know I was coming…I was seen right away in the ER…* [1]

It was important to participants that their providers responded to disclosures of STBs with genuine concern and support. Participant 4 perceived it as helpful when their provider made themself available beyond scheduled visits to offer support.

*…When my OB had found out what had happened*,* she was very quick to reach out and be like*,* ‘Hey! How are you doing? Is there anything I can do?’ *[4]

Such actions not only facilitated access to treatment, but also reduced feelings of anxiety, shame, isolation, and uncertainty for participants as they sought care for STBs.

### Treatment experiences

Regarding participants’ experiences of treatment for perinatal STBs, three major themes were identified: (1) *providers’ engagement in care*, (2) *shared decision-making between provider and patient*, and (3) *impact of perinatal-specific treatment programs*.

### Providers’ engagement in care

This theme reflects participants’ understanding of and reactions to their providers’ engagement in treatment. Sub-themes include: (1) *education in perinatal mental health*, (2) *person-centered care*, and (3) *“There was nothing we could do*.”

#### Education in perinatal mental health

Training in perinatal mental health, along with knowledge of issues specific to the perinatal period (e.g., breastfeeding), was seen as critical by nearly all participants. Prior to conception, one psychiatrist told a participant that she likely wouldn’t become depressed during pregnancy.

*[My psychiatrist] told me*,* ‘Well*,* you could probably go off your antidepressants when you get pregnant because historically*,* pregnant women don’t experience depression*,* or depression symptoms get better.’ And mind you*,* this is supposed to be the one trained professional in our area…so I was like…I don’t really believe you*,* but okay.* [8]

Others described that their psychiatrists appeared to have little knowledge of the possibility of perinatal STBs, or best practices for psychiatric prescribing among perinatal individuals.

*“The provider who switched my medication because I was pregnant—I did tell her that I was having suicidal thoughts. She told me something along the lines of*,* ‘Would you really want to hurt your baby*,* though?’ I was like*,* ‘No. That’s why I’m here telling you that I need help.’…I feel like everybody that I did talk to about it*,* as far as professionals*,* didn’t know what to say. It just seemed as though they couldn’t comprehend that somebody would have those thoughts while in this miracle time of being pregnant…it was like they were more focused on making sure that my baby’ was okay*,* and not me…I don’t feel like any of my mental health providers understood or knew how to handle somebody who is pregnant or postpartum…they were like*,* ‘I don’t know. This is what it says you can have. I’ve never had any pregnant patients.’ And it’s like*,* I’m sure you have had pregnant patients. It just seems like you have no idea how to prescribe [to] somebody who has a pregnancy…”* [11]

Access to professionals with expertise in perinatal mental health concerns positively impacted treatment experiences; participants with such access experienced less frustration, invalidation, and shame than those who perceived their providers to not have knowledge of their presenting concerns.

*I ended up getting a psychiatrist who was really*,* really focused on pregnancy and postpartum…that made a huge difference…she was very reassuring to me of how I was doing a good job—but not in a way of*,* ‘You’re doing great*,* you’re doing fine’…she was kind of practical—if your baby’s drinking breast milk*,* they’ll be fine*,* if your baby’s drinking formula*,* they’ll also be fine.* [3]

#### Person-centered care

The extent to which providers validated, respected, and listened and responded to participants as a whole person (versus dismissing or neglecting) made an important difference in how participants viewed their treatment for STBs. One provider minimized a participant’s serious mental health concerns, resulting in frustration and shame.

*[My psychiatrist] had a similar religious background and was like*,* ‘Oh*,* well*,* you have God*,* why are you depressed?…You have prayer*,* what else do you need to do? Why are you feeling like you don’t have a purpose? Your purpose is to pray.” I was like*,* ‘Okay*,* thank you?’ And then they would just change around the cocktail of meds.* [3]

Other participants described that a lack of person-centered care made them feel unimportant and devalued. One woman felt ignored and dehumanized by staff during her hospitalization, affecting treatment effectiveness.

*I’m on the phone*,* I guess quite a bit*,* because I wanna hear my daughter’s voice…she doesn’t speak*,* but I want to hear her babbling…just to feel close to her. I wanted to talk to my husband ‘cause*,* obviously*,* I was feeling very bad about our relationship and being away from him. He would call me*,* and they would tell him that I didn’t want to talk to him*,* because they felt that I was on the phone too much*,* and that it wasn’t helpful. They would tell the doctor that I didn’t want to get up for groups*,* and I didn’t want to attend therapy…But no one even told me that there was a schedule or that there was group therapy. I was sitting in my room ‘cause I thought there was nothing to do.* [11]

When person-centered care was offered, participants felt validated and cared for. When her provider went out of the way to see her quickly and provide immediate treatment for her STBs, participant 6 felt that her distress was taken seriously.

*…I love my OB ‘cause the scheduler was like…‘Okay*,* we have an opening in a month” and…my OB was like*,* “No…I need to see them sooner…something is wrong.” And so*,* she saw me on her lunch break*,* prescribed me Zoloft*,* immediately was like*,* ‘Let’s figure out what we can do for you.” And the Zoloft made such a difference. I was still so depressed*,* but it at least made me not suicidal anymore.* [6]

Another participant shared a salient story in which multiple staff members of an inpatient unit separately brought her cake on her birthday, making her feel valued and hopeful.

*That day*,* between the social worker*,* my psychiatrist*,* and my nurses*,* they all brought me five pieces of chocolate cake. And so that was all I ate all day*,* chocolate cake… They all went and spent their own money to get me chocolate cake individually*,* bringing me slices*,* and it was like in that moment—I am worthy*,* and I am worthy of living again*,* and it is okay for me to have big feelings about things that affect me. I can have those big feelings and show other people*,* and I don’t have to keep it to myself because that’s just going to hurt everyone in the long run.* [8]

#### “There was nothing we could do”

Multiple participants felt abandoned by their HCP, resulting in a lack of or delay in access to needed treatment. One woman with postpartum psychosis was discharged from the hospital while still in active crisis.

*I went back to the hospital on a Saturday… she sent me home on Tuesday*,* still in active psychosis. And she just said that there was nothing that they could do for me there*,* which is the most mind-boggling thing I’ve ever heard in my life…She said that they were a stabilizing unit and so because I was stable*,* they had to send me home. I was like*,* I don’t think that this is stable…* [1]

A similar sentiment was shared by participant 8, whose psychiatrist failed to provide additional treatment options after she shared an aborted suicide attempt, further increasing feelings of abandonment and isolation.

*I actually tried driving into traffic and then something stopped me*,* and I drove home. The next day after that*,* I saw my psychiatrist*,* and I told her about it*,* and I asked her what we could do ‘cause I was at the highest of my antidepressants that I could be at. She basically told me there was nothing we could do for another month and a half because I was too early in my pregnancy. She just wrote it in my records that I was having a difficult time and sent me home and I remember crying the entire way home*,* and at that time*,* my doctor was 45 minutes away from my house. So*,* I just cried all the way home thinking—she just gave me permission to just drive home and drive into traffic—and having intrusive thoughts the entire way home of ‘Just turn*,* just turn.’* [8]

Others felt that their providers neglected maternal well-being in favor of survival of pregnancy, relinquishing active responsibility for mental health.

*The focus was*,* deliver this healthy baby. And then when she was born*,* it was like*,* well*,* you’re a grown-up*,* take care of yourself. And*,* of course*,* I can take care of myself*,* but it was just so fucking hard. And I felt like my providers…just didn’t honor domestic violence*,* I guess—refused to give me space or time or resources*,* or check-in. It was really a sense of*,* ‘You’re on your own.’* [5]

Few shared experiences of their treatment team exhibiting persistence in ensuring effective treatment was provided, but when discussed, they were appreciated and felt validated as a result.

### Shared decision-making between provider and patient

This theme reflects the perceived importance of autonomy and collaboration in decisions about treatment for perinatal STBs, particularly as it pertains to psychiatric medications. This was succinctly described by participant 5 when she said, “*I think a conversation [about medication] would’ve been helpful—not ‘Here’s your prescription*,* bye.’…but ‘Let’s talk about this.’”* Another participant shared that, despite sharing her previous medication trials with her psychiatrist, was taken off an effective medication regimen during pregnancy, ultimately leading to a worsening of her mental health symptoms.

*When I moved*,* the doctor said that I can’t be on [my medication]*,* that it’s a horrible idea to stay on it—even though I looked it up after*,* and there was nothing really that said that it was bad for you…she moved me to a medication that I told her I had tried in the past and it didn’t work for me…I was originally on lurasidone*,* and then she switched me to olanzapine and sertraline…She said those are the ones that she knew were safe for pregnancy and that’s all she would give me*,* and if I didn’t like it*,* I could go somewhere else…I actually started crying in her office ‘cause I was like*,* ‘I don’t know where else to go.’ I don’t have another doctor near me…I kept seeing her through the remainder of my pregnancy and she kept me on that medication. And they said that I wasn’t doing well on the medication—which I already told them [would be the case].* [11]

Others felt that they were active participants in their treatment; one participant shared an appreciation for having her psychiatrist’s support to independently increase her medication on weekends.

*[My psychiatrist] listens really well…she just is a good advocate and listens and understands mental health for women…she’s very validating*,* she listens*,* she’s empowering*,* like*,* ‘If you are on a weekend and you think you need to increase your dose to this amount*,* you go ahead and do that. You just call me Monday*,* and I’ll have your prescription updated.’ She really lets me make that choice for myself and for my body on what I think is best*,* so she’s great.* [10]

### Impact of perinatal-specific treatment programs

Many attended IOPs or were hospitalized as part of their treatment for STBs. This theme reflects the differences between experiences in perinatal-specific treatment programs versus those not targeted toward perinatal individuals. Generally, perinatal-specific programs were experienced as supportive and validating. Participant 8 attended a perinatal-specific IOP in which she was able to connect with other patients experiencing similar challenges.

*…There were*,* depending on the week*,* between 6 and 10 women in the group in their outpatient program*,* pregnant or postpartum. And that also was really supportive and empowering. ‘Cause I’m sitting there like—I feel so judged*,* and everyone’s going to judge me and tell me I’m a bad mom because of all of the things that are happening*,* and they were all also going through similar things.* [8]

Similarly, participant 13 was hospitalized in an inpatient unit specifically for perinatal individuals, which provided a sense of hope.

*One of the things that was…most helpful about the [program] was that they had a book of letters from moms when they first got there versus when they got out—when they were being discharged. And seeing people be like*,* ‘It’s fucking different*,*’ gave me hope. If all of these—and there were a lot—if all of these women felt even a little bit better*,* that means I can do it too*,* right? And it was validating to know that I’m not the only [one]…* [13]

However, she also experienced challenges in her hospitalization. While the program was specifically for perinatal individuals, construction resulted in units mixing, and a sense of frustration and invalidation.

*“They were remodeling the perinatal unit*,* so I was pushed into the normal unit*,* and because they just wanted to fill beds*,* even though I was in the perinatal unit*,* they were putting just any woman who was of childbearing age. So*,* even though all the groups and activities and therapies were all tailored to perinatal women*,* 85% of the women I was in there with weren’t moms. So*,* they were making adjustments for them*,* and I was like*,* no…Their programming should be with the other people. Do not devalue my experience here because you’re letting other people in.* [13]

A similar sentiment was shared by participant 3, who attended a non-perinatal-specific IOP during her pregnancy, and experienced shame from harmful comments from other adult patients.

*…It wasn’t tailored to pregnant people…It was just a mixed bag of different people that were experiencing depression. But even in that situation*,* I just felt like I was the most depressed of everyone—I don’t know why—but that I had the least reason to be…I would also compare myself to the other people in the group and be like*,* this person’s going through this and this situation*,* like*,* why am I here? What’s wrong with me?… Even other people in the group would be like*,* ‘Having your baby is such a blessing’ and*,* you know*,* ‘It’s going to be so great’…You can’t do a broad brush and include pregnant people with everybody else*,* ‘cause it’s not the same.* [3]

A common opinion amongst women who were hospitalized for their perinatal STBs was that being away from their child(ren) was among the hardest parts of their hospitalizations. Participant 11, who was hospitalized in a non-perinatal-specific unit, felt the environment was not conductive to her recovery.

*I felt like being in the hospital was way worse for me because I was constantly away from my daughter. I couldn’t see her. I could barely hear her on the phone…And I just felt like the staff really just didn’t want to be bothered…It felt like—almost like prison*,* or what I’ve heard other people talk about jail feeling like. But also*,* I felt like they weren’t trying to get me to have care*,* they were just trying to keep me there for no reason…no doctor came to talk to me for a couple of days.* [11]

Though not all participants in our sample received treatment for their STBs, those who did described more positive treatment experiences when their providers were knowledgeable about perinatal mental health issues, validating, and persistent in ensuring they received the support they needed, when they felt like they were active participants in treatment decisions, and when higher levels of care (i.e., IOPs, inpatient units) were attentive to their perinatal-specific needs.

## Discussion

The present study drew on the lived experiences of 13 individuals with histories of perinatal STBs to identify themes pertaining to screening and treatment, which has largely been unaddressed in the literature to date, particularly in the U.S. The sample, though limited in socioeconomic diversity, was relatively racially diverse compared to existing literature on this topic, and resided in various regions of the U.S. and Canada, providing accounts of their experiences across several domains. Our findings point to several important future directions for both research and clinical practice.

Within respect to screening experiences, multiple salient themes were identified. Our findings suggest that current perinatal mental health screening practices may lack specificity for STBs and may not occur routinely or frequently, leading to gaps in opportunities for HCPs to identify and treat individuals at risk for suicide. It is important to note that these gaps may be shaped by the type of setting in which the screening occurs (e.g., obstetric, mental health), as time constraints, risk management processes, and other factors that vary across contexts may have important impacts on patient experiences. Notably, while widely used tools such as the EPDS are recommended by several national clinical practice guidelines and professional organizations [15–17], results suggest that these tools alone may be insufficient in capturing the full spectrum of perinatal STBs. Our findings also suggest that *how* screening occurs is just as important as *whether* it happens; screening was often perceived to be inauthentic or ingenuine. The manner in which such screening tools are considered by patients to be as equally important as their effectiveness. To reduce maternal mortality due to suicide, clinical practice must incorporate proactive, empathetic screening for perinatal STBs at multiple timepoints over the course of pregnancy and postpartum. It is also worth noting that screening for perinatal STBs varies by cultural and national contexts, and these differences likely shape patient experiences in important ways. In the U.S., the absence of clear guidelines regarding screening for perinatal STBs, insurance-based healthcare access, and fragmentation between obstetric and mental healthcare may further compound the gaps identified by participants.

Participants also held salient attitudes towards disclosure of STBs, resulting in decisions about whether or not to tell the truth during screening and therefore whether or not treatment was sought. Many of our participants held intense fears that they would be negatively evaluated as a mother, have their baby taken away, or be involuntarily hospitalized, consistent with prior U.K.-based qualitative findings [25]. Others felt that disclosing STBs would not lead to meaningful support and could cause more harm. Still others chose not to disclose their STBs because they did not specifically identify with the word “suicidal,” aligning with previous work that found standard suicide screening language to be alienating, stigmatizing, or confronting [24]. Multiple factors contribute to the decision of whether or not to disclose STBs in healthcare settings; screening approaches should thus be sensitive to language, normalize STBs using validating and nonjudgmental language, and address fears of potential punitive consequences.

Our findings also reinforce that screening for STBs without action is perceived to be insufficient. Even when participants made the difficult decision to share their STBs with a HCP during screening, many felt as if there was a lack of appropriate follow-up after, or that their STBs were dismissed once providers confirmed that they were already engaged in treatment. Clear follow-up protocols (e.g., warm hand-offs, referral lists to programs providing perinatal mental health services) may help to ensure that individuals experiencing perinatal STBs receive immediate and appropriate support.

Participants raised several meaningful concerns related to their experiences in treatment for STBs. Providers themselves played a pivotal role in how treatment was perceived; for instance, participants found their treatment to not only be more effective but also more validating when their clinician had prior knowledge or education in perinatal mental health. Relational quality (e.g., empathy, person-centeredness) also resulted in more validating experiences in treatment for perinatal STBs, consistent with U.K. qualitative work in the area of screening [25]. Some providers were also described to have “given up,” further contributing to a sense of hopelessness. Perceived abandonment, dismissal, invalidation, and lack of training in perinatal mental health caused unnecessary delays in participants recovering and provide important directions for not only current clinical care but also training of future mental health and obstetric providers. Shared decision-making between provider and patient was found to be a desired (though not always experienced) aspect of treatment experiences. Our findings highlight the importance of collaboration between providers and patients when it comes to decisions regarding treatment. Treatment of perinatal STBs should emphasize autonomy and transparency to build trust and facilitate recovery.

Though not all of our participants received a higher level of care (i.e., IOPs, inpatient units), a salient theme was identified regarding the impact of perinatal-specific programs. Among those that received treatment from an IOP or inpatient hospitalization, individuals receiving care from programs tailored to perinatal populations were seen as more acceptable and desirable compared to those who attended general adult programs. This finding aligns with prior work emphasizing the unique hospitalization needs of individuals with perinatal mental health conditions (e.g., postpartum psychosis), compared to other adults [34–36]. However, it is important to note that while such tailored programs are considered best practice in other parts of the world (e.g., Mother-Baby Units in the U.K.) [37], they are rare and difficult to access in the U.S [38]. Future research will be important to delineate the positive long-term outcomes following receipt of treatment in specialized perinatal mental health inpatient or day treatment programs.

The present study has several limitations. While our sample size is considered appropriate for qualitative analyses [39, 40], it is possible that the sample composition may limit the study’s generalizability. We acknowledge the relative lack of socioeconomic and geographic diversity (e.g., highly educated, primarily urban/suburban, with access to healthcare services), which has important implications for the applicability of findings to populations most affected by perinatal suicide risk. Socioeconomic status has been identified across several reviews to associated with perinatal STBs [41–44]. Future research will be critical to exploring the perceptions of screening and treatment for perinatal STBs among more socioeconomically vulnerable populations, who face heightened barriers to care. Additionally, while the vast majority of participants lived in the U.S., one participant resided in Canada; given differences between Canadian and U.S. healthcare systems, findings should be interpreted as primarily reflecting the U.S. context.

Second, we elected to use the umbrella term of perinatal STBs to reflect the sample’s heterogenous experiences, including passive SI, active SI, and aborted suicide attempts. Screening, risk assessment, and treatment likely differ substantially across the STB spectrum, particularly with respect to involvement of crisis services and higher levels of care. Our sample was limited in that few participants engaged in suicidal behaviors. Thus, our findings may not be applicable to those experiencing more acute suicide risk in the perinatal period. Future research would benefit from distinguishing how screening and treatment experiences vary across levels of perinatal suicide risk.

Finally, as our inclusion criteria allowed for participants who experienced perinatal STBs at least six months prior to enrollment, recall and reconstruction bias may have shaped how their experiences were shared during the interview process. It is important to acknowledge that subsequent recovery trajectories, which were not comprehensively explored in this study, may have influenced participant narratives.

We also identify several strengths of the present research. To our knowledge, this is the first investigation into the specific healthcare experiences (i.e., screening and treatment) of individuals with perinatal STBs. Our exploratory qualitative design allowed not only for an in-depth investigation of a sensitive, complex, and underreported issue, but also generated rich insights that likely would not have been uncovered otherwise (e.g., with traditional quantitative methods). Further, our semi-structured approach facilitated the collection of descriptive narratives in which participants were able to share aspects of their experiences most meaningful or salient to them. An additional strength of the study is the relatively racially diverse and primarily U.S.-based sample and recruitment of individuals from across diverse geographical regions. Existing qualitative research on perinatal STBs is almost exclusively based in the U.K. and Australia with predominantly White samples. Given the vastly different healthcare systems in the U.S. versus other countries, and due to salient differences in healthcare experiences due to racism, socioeconomic disadvantage, and other structural inequities, continued examination of perinatal STBs with global diverse samples is critical.

## Conclusions

Findings from this study highlight critical gaps in screening for and treatment of perinatal STBs. Implementing routine screening and comprehensive follow-up and improving treatment experiences are essential for ensuring individuals with perinatal STBs receive the care they need. Given contribution of perinatal suicide to the U.S.’s persistently high rates of maternal mortality, such action is vital for the well-being of mothers and families.

## Electronic Supplementary Material

Below is the link to the electronic supplementary material.

Supplementary Material 1

## Data Availability

The datasets used and analyzed as part of this study are available from the corresponding author on reasonable request.

## Abbreviations

HCP: Healthcare provider
IOP: Intensive outpatient program
PEARS: Perinatal Experiences and Assessment of Recovery from Suicidality
SI: Suicidal ideation
STB: Suicidal thoughts and behaviors
UK: United Kingdom
US: United States
