# Supplementary material for: “I just held it to myself”: screening and treatment experiences of individuals with perinatal suicidal thoughts and behaviors
Source: BMC Psychiatry. 2026 May 2;26:482. doi: 10.1186/s12888-026-08129-3 (PMC13289127; doi:10.1186/s12888-026-08129-3)
Supplement: Supplementary file 1 — Supplementary Material 1 [file 12888_2026_8129_MOESM1_ESM.docx]

**Supplementary Information**

PEARS Individual Interview Guide

*Note: Words in bold/italics refer to directions and notes for the interviewer, and are not to be read verbatim to the participant.*

As you know, the purpose of this study is to learn more about people’s experiences with suicidality during pregnancy or postpartum – an issue that isn’t talked about very much, but is very important to address in order to improve maternal mental health, and by extension, the health of babies and families. We’re also interested in learning more about what types of services or treatments can help individuals with suicidality during this period of time. I very much appreciate your taking the time to talk with me about this topic.

Remember that you can stop the interview at any time if you don’t feel comfortable. Are you ready to begin? [*Begin recording*]

**Introduction:** [*Get to know participant; increase comfort with interview*]

- Tell me a little bit about yourself.
- Are you currently working?
  - *If yes*: What do you do for work?
- What do you like to do in your free time? [*Probe for: hobbies; pleasant activities*]
- Tell me about your family. [*Probe for: family composition; marital status; number/ages of children 🡪 number of experiences with perinatal suicidality; length of time since episode*]

**Onset of Suicidality**: [*If more than one perinatal experience, refer to the most recent experience*]

- Think back to the first time during pregnancy or postpartum that you thought you would be better off dead or of hurting yourself in some way. Can you tell me what that was like for you?
  - At what point did you begin to think about suicide during your pregnancy/after the birth of your child?
  - What was going on in your life at the time?
    - Tell me more about any triggers or events that could have contributed to these thoughts.
  - How would you describe your mood at the time?
- What reasons did you have for thinking about wanting to die or killing yourself?

*If participant describes ongoing issues related to suicidality, assess for active vs. passive suicidal ideation. Probe for intent, plan, access to means, and current supports.* ***Refer to safety protocol per clinical judgement.***

**Coping Mechanisms**:

- Tell me how you dealt with the thoughts about harming yourself or dying.
  - How did you cope with this experience?
- Did you tell someone about these thoughts?
  - *If yes*: Who was this person? Why did you choose to tell that person? How much did you share with them?
  - *If no one*: Why did you choose not to tell anyone?
- What coping strategies were helpful for you at the time?
  - Were there any that weren’t useful for you?
- What gave you hope during that time?

**Treatment Seeking & Experiences:**

- Did any provider ask you about thoughts of harming yourself during your prenatal or postpartum visits? [*Probe for: screening/questionnaires; provider-client relationship; provider responses; respectful care*]
  - *If yes*: What was that like?
  - *If no*: Would it have been helpful for someone to have asked?
- Did you seek medical care for your thoughts about harming yourself or dying?
  - *If yes*: Why did you choose to seek help?
    - What was your experience seeking help for suicidality?
    - What was your experience with treatment for suicidality?
  - *If no*: Why did you choose not to seek help? [*Probe for: stigma; cultural norms*]
- What would have made it easier or more likely for you to seek care?
  - What would have made it harder or less likely for you to seek care?
  - [*Probe for: system-level factors; insurance; childcare; wait times for appointments]*

**Intervention Preferences**:

- Currently, there are no specific interventions (prevention or treatment) for pregnant or postpartum individuals that address suicidality. Why do you think that is? [*Probe for: stigma; shame; guilt; myths about motherhood; cultural norms*]
- Looking back on your situation, what would you have found helpful in an intervention to support women with these thoughts?
  - Would you have preferred an individual or group setting?
  - Would you have preferred to receive treatment in-person or via telehealth?
  - Which providers would have been helpful?
- What topics should be covered in a mental health intervention for women who may be at risk for having these thoughts?
  - What should a prevention program look like?
  - What do you think could have helped prevent you from having this experience?

**Ending Questions**:

- What would you say to other pregnant or postpartum women who are experiencing suicidal thoughts?
- What gives you hope today?
- Is there anything we didn’t ask about your experience that you think would be important for us to know?
